# Nanosphere Self-Assembly Imaging Systems and Defect Detection Algorithms for Self-Assembled Structures: A Review

**DOI:** 10.3390/nano16140890

**Published:** 2026-07-20

**Authors:** Qihang Liu, Yuang Chen, Qingwei Zhou, Jinbao Jiang, Fang Luo, Fan Wu, Chucai Guo, Zhihong Zhu, Dan Chen

**Affiliations:** 1 College of Advanced Interdisciplinary Studies & Hunan Provincial Key Laboratory of Novel Nano-Optoelectronic Information Materials and Devices, National University of Defense Technology, Changsha 410073, China; 18859967097@163.com (Q.L.); chenyuang0712@163.com (Y.C.); zqw2100@163.com (Q.Z.); jiangjinbao21@nudt.edu.cn (J.J.); luofang2013@163.com (F.L.); 13808490405@139.com (F.W.); gcc_1981@163.com (C.G.); zzhwcx@163.com (Z.Z.); 2 Nanhu Laser Laboratory, National University of Defense Technology, Changsha 410073, China

**Keywords:** self-assembled nanospheres, defect detection, imaging systems, correlative microscopy, machine learning, deep learning

## Abstract

Self-assembled nanosphere structures are widely used as bottom-up platforms for ordered micro- and nanostructures, with applications in photonic crystals, sensing platforms, functional coatings, drug delivery, and nanosphere lithography. Their performance and reproducibility depend on structural order, packing density, interparticle spacing, and defect density and distribution. Thus, reliable imaging and quantitative defect detection are needed for quality evaluation and process optimization. This review provides an overview of defect characteristics, imaging systems, and defect detection algorithms for self-assembled nanosphere structures. It first introduces representative zero-, one-, two-, and three-dimensional assemblies, followed by a summary of common defects, including vacancies, interstitial particles, dislocations, grain boundaries, stacking faults, voids, and cracks. Optical microscopy, electron microscopy, atomic force microscopy, scanning near-field optical microscopy, and correlative techniques are compared in terms of resolution, field of view, temporal resolution, contrast mechanism, in situ capability, and compatibility with feedback control. Algorithmic approaches are also reviewed, encompassing classical image processing, machine learning, and deep learning, along with their applications in segmentation, localization, classification, and high-throughput analysis. Overall, reliable defect inspection requires integrated workflows. These workflows should combine appropriate imaging systems, image quality control, transferable algorithms, standardized datasets, and closed-loop feedback.

## 1. Introduction

Self-assembly of nanospheres has emerged as a powerful bottom-up strategy for constructing ordered micro- and nanostructures through local interactions among building blocks, including van der Waals forces, electrostatic interactions, hydrogen bonding, hydrophobic interactions, and capillary forces [1,2]. Compared with conventional top-down fabrication, this approach offers distinct advantages in parallel processing, structural tunability, material versatility, and scalable pattern formation [3]. As a result, self-assembled nanosphere structures have been widely explored in photonic crystals, surface-enhanced Raman scattering substrates, optical sensors, drug delivery systems, functional coatings, and nanosphere lithography [4,5]. In these applications, the collective properties of the assembled structures are determined not only by the composition, size, and surface chemistry of individual nanospheres, but also by their spatial arrangement, long-range ordering, packing density, and interparticle spacing. However, structural imperfections are inherently unavoidable because of particle size dispersity, surface chemical heterogeneity, substrate effects, nonequilibrium assembly kinetics, and drying-induced stresses. Defects such as vacancies, interstitial particles, dislocations, grain boundaries, multilayer stacking, voids, and cracks can disturb periodic order, modify local optical or mechanical responses, reduce signal reproducibility, and ultimately limit device performance. Therefore, reliable imaging and quantitative defect detection are essential for understanding assembly mechanisms, evaluating structural quality, and guiding process optimization.

Despite extensive progress in nanosphere self-assembly and microscopic characterization, several challenges remain in the inspection and analysis of self-assembled structures. First, no single imaging modality can fully satisfy the diverse requirements of defect inspection. Optical microscopy provides rapid, nondestructive, and large-area observation; yet, its spatial resolution is insufficient for resolving nanoscale packing defects. SEM and TEM offer substantially higher spatial resolution and enable direct visualization of local morphology, lattice order, and internal structure. Nevertheless, they may suffer from limited imaging throughput, vacuum requirements, beam-induced damage, and restricted compatibility with dynamic liquid-phase processes. AFM, SNOM, and other complementary imaging techniques can provide three-dimensional topography, local optical-field distributions, chemical specificity, or dynamic information, yet they are often limited by small field of view, slow scanning speed, or complex operation. Recent studies on automated self-assembly platforms and correlative imaging strategies further indicate that future imaging systems need to move from static post-characterization toward in situ, multimodal, and feedback-capable observation [6,7]. Second, defect analysis remains algorithmically challenging. Traditional image processing methods, including threshold segmentation, edge detection, and frequency-domain analysis, are efficient and interpretable, while their performance is often sensitive to image contrast, noise, illumination variation, and manually selected parameters [8]. Machine learning methods improve classification and pattern recognition by combining handcrafted features with statistical models. Despite this, their generalization is constrained by the quality and representational capacity of predefined features [9]. Deep learning methods can learn hierarchical features directly from microscopy images and have shown strong potential for defect segmentation, localization, and high-throughput nanostructure analysis; nevertheless, they usually require large annotated datasets and may exhibit limited robustness across different materials, imaging conditions, magnifications, and defect morphologies [10,11]. These limitations indicate that defect inspection of self-assembled nanosphere structures should be considered as an integrated problem involving defect characteristics, imaging-system selection, image quality, algorithm design, and quantitative evaluation.

In this review, we provide a systematic overview of imaging systems and defect detection algorithms for self-assembled nanosphere structures. We summarize representative self-assembled architectures and common defect types, emphasizing how different defects influence structural integrity and functional performance. We then review major imaging modalities used for nanosphere self-assembly, including optical microscopy, electron microscopy, atomic force microscopy, scanning near-field optical microscopy, and other complementary techniques. Particular attention is given to spatial resolution, field of view, temporal resolution, image contrast, in situ capability, intelligent imaging, and multimodal correlative microscopy. Subsequently, we discuss defect detection algorithms from three methodological perspectives: classical image processing methods, machine learning approaches, and deep-learning-based segmentation and object detection frameworks. By linking defect characteristics with imaging requirements and algorithmic strategies, this review aims to clarify the current state of the field, highlight remaining technical bottlenecks, and provide guidance for the development of more automated, quantitative, and feedback-driven inspection workflows for self-assembled nanosphere systems.

## 2. Defect Characteristics and Inspection Requirements of Self-Assembled Structures

Self-assembly of nanospheres is a spontaneous process that does not require external energy input. It relies on weak local interactions such as van der Waals forces, hydrogen bonds, hydrophobic interactions, and electrostatic interactions between nano-building blocks (NBBs), thereby spontaneously forming thermodynamically more stable ordered structures. This bottom-up approach has shown broad application prospects in fields such as photonic crystals, sensors, drug delivery, and functional coatings. However, size dispersity, surface chemical heterogeneity, and the uncontrollability of the assembly kinetics process inevitably introduce structural imperfections such as lattice defects, vacancies, dislocations, and polycrystalline domain boundaries, which significantly affect their structural integrity and functional performance. This section mainly reviews the representative dimensional architectures of nanosphere assemblies and the corresponding defects.

### 2.1. Typical Self-Assembled Structures

The dimensionality of nanomaterials is a key factor determining their physicochemical properties and application potential. Self-assembly strategies have enabled the construction of architectures spanning from zero to three dimensions. However, such dimensional classification should not be considered purely from a structural perspective, because the final architecture is also closely related to the kinetics of nucleation, layer growth, island formation, and defect generation.

Although the colloidal nanosphere assemblies discussed in this review are not identical to atom-by-atom epitaxial films, the classical Frank–van der Merwe, Volmer–Weber, and Stranski–Krastanow growth modes provide a useful kinetic framework for understanding how 0D islands, 2D layers, and layer-plus-island structures form. In the Frank–van der Merwe mode, favorable wetting and low interfacial mismatch promote layer-by-layer growth; in the Volmer–Weber mode, weak wetting favors direct island nucleation; and in the Stranski–Krastanow mode, an initial wetting layer is followed by three-dimensional island formation once strain or interfacial energy accumulates [12]. These concepts are particularly relevant to 0D and 2D nanostructures because nucleation density, island size, layer continuity, and roughness are strongly coupled to substrate interactions, lattice or interfacial mismatch, surface diffusion, and deposition conditions. From this kinetic perspective, the representative self-assembled architectures discussed below can be viewed not only as structures with different dimensionalities, but also as outcomes of different pathways of nucleation, spreading, coalescence, and defect generation.

**Figure 1:**
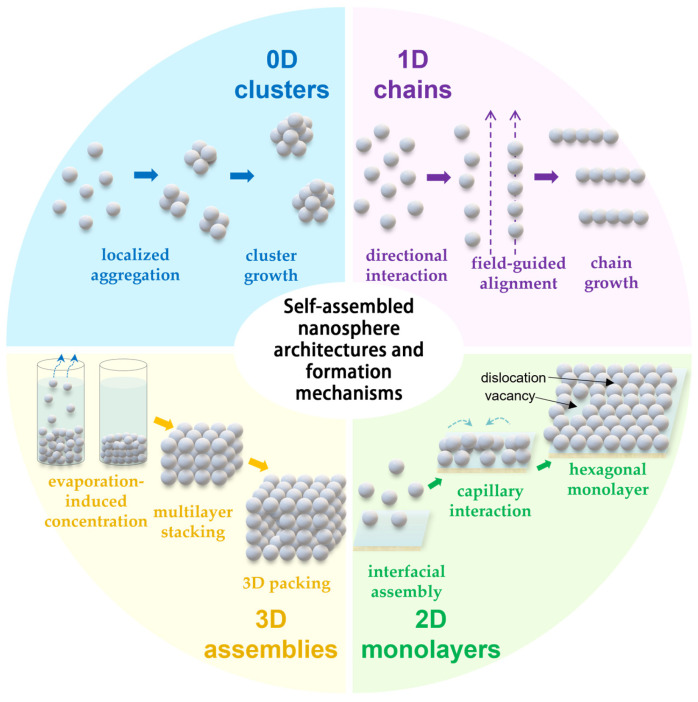
General schematic illustration of representative self-assembled nanosphere architectures and their formation mechanisms. Zero-dimensional clusters formed through a nucleation-dominated pathway, including dispersed nanospheres, localized nucleation, aggregation, and island growth; one-dimensional chain-like assemblies generated by directional interparticle interactions or field-guided alignment; two-dimensional monolayers formed by interfacial assembly and capillary-driven organization into hexagonal close-packed arrays, with typical defects such as vacancies, dislocations, and grain boundaries; three-dimensional assemblies produced by evaporation-driven concentration or sedimentation followed by multilayer stacking and three-dimensional packing.

A general schematic summary of representative self-assembled nanosphere architectures and their dominant formation pathways is shown in Figure 1.

#### 2.1.1. Zero-Dimensional (0D) Structures

Zero-dimensional structures are discrete nanoscale units confined in all three spatial directions, including spherical nanoparticles, quantum dots, and nanoclusters assembled. They commonly serve as fundamental units for building higher-order structures. For example, monodisperse silica or polymer nanospheres can be assembled into macroscopic colloidal crystals via evaporation-induced assembly, effectively acting as artificial atoms. Recent studies have expanded the functional scope of 0D assemblies. Wang et al. utilized an ice–liquid interface strategy to direct the stacking self-assembly of zero-dimensional polymer nanospheres. The results demonstrated that the internal free volume network enables efficient ion-selective transport without continuous pores [13]. Wang et al. induced the self-assembly of zero-dimensional room-temperature phosphorescent SiO_2_ nanospheres into photonic crystals via evaporation, demonstrating angle-dependent structural color, fluorescence, and phosphorescence signals. Such multifunctional 0D assemblies provide promising avenues for multimodal optical sensing and anti-counterfeiting device fabrication [14].

#### 2.1.2. One-Dimensional (1D) Structures

One-dimensional structures extend markedly in one direction while being confined in the other two, forming nanowires, nanotubes, and nanorods. The assembly of 1D structures commonly depends on anisotropic interactions. For example, Janus nanospheres or high-aspect-ratio nanorods can assemble into one-dimensional chain-like structures through end-to-end or side-by-side connections. This arrangement provides unique applications in photonics and electronics. The chains assembled from metal nanoparticles can produce strong plasmonic coupling effects and serve as platforms for constructing ultra-sensitive molecular sensors [15]. A recent investigation on nanorods indicates that the liquid crystal one-dimensional nanorod structures prepared by Wang et al. enable fast orientation tuning under an applied magnetic field. This alters their optical transmission and reflection properties and has been successfully applied in magnetically responsive display devices [16].

#### 2.1.3. Two-Dimensional (2D) Structures

Two-dimensional structures extend across a plane, forming nanosheets, monolayers, or two-dimensional superlattices. Large-area, monolayer, and ordered nanosphere arrays are typically produced by interfacial self-assembly. The nanosphere lithography technique developed by the groups of C. Haynes and R. P. Duyne employs a monolayer of nanospheres arranged in a two-dimensional hexagonal close-packed lattice as a mask. Subsequent deposition or etching processes enable the large-scale fabrication of periodic nanostructure arrays on substrates [17].

Recent studies have further extended this topic from ideal hexagonal monolayers to quantitative quality evaluation and short-range ordered nanohole structures. Domonkos and Kromka reviewed nanosphere-lithography-based fabrication of spherical nanostructures and emphasized the importance of verifying hexagonal symmetry and defect configurations by image analysis, while the corresponding HEXI-based image analysis strategy was further detailed for distinguishing hexagonally ordered and defective spherical nanostructures [18,19]. In addition, Cesaria and co-workers investigated short-range ordered gold nanohole arrays and showed that their optical transmittance features are strongly affected by local coordination geometry, packing evolution, and short-range periodicity rather than by a simple periodic lattice constant alone [20,21]. In advanced applications, Elif Lulek et al. demonstrated that large-area monolayer two-dimensional colloidal crystals prepared via gas/liquid interface self-assembly, when coated with a gold film, serve as high-performance substrates. These substrates markedly enhance surface-enhanced Raman scattering (SERS) signals, facilitating trace-level substance detection [22].

#### 2.1.4. Three-Dimensional (3D) Structures

Three-dimensional structures feature complex nanoscale architectures across all three spatial dimensions, including colloidal photonic crystals, porous frameworks, and hierarchical structures. The assembly of these structures generally involves intricate, multi-force driving mechanisms. In three-dimensional colloidal systems with relatively large particle sizes, self-assembly must maintain long-range order within dynamic evaporative nonequilibrium flow fields while overcoming gravitational sedimentation, which occasionally requires adjustment of environmental parameters. Owing to the complex interplay of multiple driving forces in three-dimensional structures, their applications are widespread in advanced research areas. For example, Lei Wen and co-workers utilized boron-doping-induced self-assembly to construct a three-dimensional interconnected SiOCB hollow porous nanosphere framework, effectively mitigating volume expansion in electrode materials and significantly improving the cycling stability of lithium-ion batteries. Nevertheless, bridging these lab-scale three-dimensional architectures to scalable manufacturing tends to multiply defects at every length scale, a challenge that remains largely unresolved [23].

### 2.2. Common Defect Types and Their Effects

**Figure 2:**
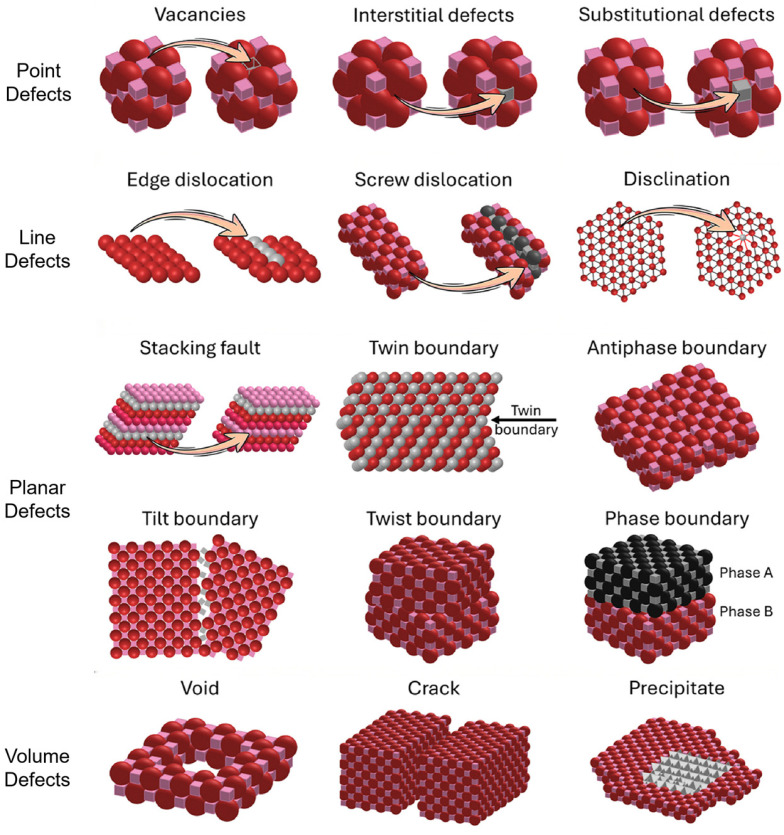
Schematic representation of defects in a superstructure, including point defects (0D), line defects (1D), planar defects (2D), and volume defects (3D). Reprinted with permission from Ref. [24]. 2025, Fonseca, J. et al.

Nanosphere self-assembled structures exhibit unique functional characteristics in many fields due to their inherent structural features. However, various defects deviating from the ideal ordered structure are difficult to avoid during the actual assembly process, which profoundly affects the performance of the assembled materials. Javier Fonseca, in his research on superstructures, systematically classified defects into four main categories based on geometric dimensions: point, line, planar, and volume defects. The following subsections discuss each category and its impact on physicochemical performance (Figure 2) [24].

#### 2.2.1. Point Defects

Point defects are zero-dimensional localized lattice distortions. Typical examples include vacancies (missing nanospheres at lattice points), interstitial defects (extra nanospheres located at non-lattice sites), and substitutional defects (nanospheres of different sizes or properties occupying the original lattice points). These defects typically originate from the polydispersity of size, local kinetic fluctuations, or substrate heterogeneity. Despite the small scale, point defects can cause significant degradation of optical performance through localized refractive index discontinuities. Canalejas-Tejero et al. observed that single-particle vacancies in two-dimensional hexagonally close-packed polystyrene and silica nanosphere arrays disrupt the periodic dielectric distribution. That further generates incoherent scattering background, significantly reducing the photonic bandgap depth and structural color saturation. This investigation demonstrates that even a single missing sphere can bring about point defects and, therefore, measurably alter collective optical behavior [25].

#### 2.2.2. Line Defects

Line defects are one-dimensional defects, primarily dislocations (edge and screw dislocations). They typically form due to stress accumulation during crystal growth or misorientation upon the coalescence of two nuclei at a low-angle boundary. Line defects have a pronounced dual effect on the mechanical properties of colloidal crystals. Svetlizky et al. used high-speed, three-dimensional confocal real-time microscopy to show that the nucleation, slip, and multiplication of line dislocations intersect within a three-dimensional network. This interaction triggers sudden, sharp plastic relaxation, rapidly disrupting the crystal’s long-range order [26]. On the other hand, Kim et al. later observed opposite mechanical effects in shear-loaded hard sphere colloidal crystals. After the dislocation density increased, stable dislocation tangles and locks formed via entanglement and interlocking, resulting in strain hardening rather than softening. This dichotomy highlights that the mechanical role of dislocations is highly dependent on the load regime and particle interaction potential [27].

#### 2.2.3. Planar Defects

Planar defects are two-dimensional defects, including grain boundaries (interfaces between grains with different orientations), stacking faults (errors in the atomic layer stacking sequence), twin boundaries, and phase boundaries (interfaces between different phases). An investigation embeds a two-dimensional organic light-emitting planar defect layer within opal structures self-assembled from PS nanospheres. This artificial planar defect can open a designable transmission window within the photonic bandgap and enhance emission, enabling active control over the internal optical behavior. Thus, planar defects represent a double-edged sword: they are deleterious to passive photonic devices yet valuable for active optical design when precisely positioned [28].

#### 2.2.4. Volume Defects

Volume defects are three-dimensional macroscopic defects, including voids, cracks, and large impurity inclusions. These defects usually arise from improper process control, such as uneven solution concentration leading to internal stress mismatch between capillary forces and substrate adhesion during drying. The destructive effects of volume defects are more pronounced. Voids act as stress concentrations under tensile stress induced by solvent evaporation. Nucleating cracks form a continuous mechanical failure pathway from microscopic vacancies to macroscopic fractures, ultimately compromising the structural integrity of the assembly [29]. Moreover, when self-assemblies are used as SERS substrates or catalytic supports, cracks and pores cause highly uneven distribution of active sites, resulting in poor signal reproducibility or reduced catalytic efficiency.

While the geometric classification of defects, from point to volume, provides an intuitive framework, real-world colloidal assemblies often exhibit correlated, multiscale defect clusters. Dislocations pile up at grain boundaries, grain boundaries initiate cracks, and vacancies cluster into voids. Few studies have quantified the cooperative effect of mixed-dimensionality defects on collective optical or mechanical properties. Multiscale modeling correlated with in situ imaging will be essential to decode these interactions.

## 3. Imaging Systems for Micro-Nanosphere Self-Assembly

Imaging serves as the critical bridge between physical assembly processes and computational analysis. The choice of imaging modality determines the detectable defect types, spatial scales, temporal resolution, and sample compatibility. Consequently, selecting an appropriate system and optimizing its critical parameters is essential for reliable nanoscale characterization. The integration of real-time imaging with intelligent feedback systems further represents a transformative direction for the field.

### 3.1. Optical Microscope

Optical microscopes remain the preferred tool for observing self-assembly dynamics and macroscopic defects due to the non-invasive nature, ease of use, and high frame rates. McGlade et al. used optical microscopy to monitor how substrate pre-patterning guides ordering in polystyrene microsphere arrays and documented a measurable reduction in defect density [30]. In a recent study on metal–semiconductor nanocluster assemblies, optical microscopy is first employed to observe rainbow interference patterns in centimeter-scale films. Subsequently, TEM was used to reveal the nanoscale filamentous subunits forming these structures. This sequential approach exemplifies the value of optical microscopy for rapid screening and macroscopic localization prior to high-resolution interrogation [31].

**Figure 3:**
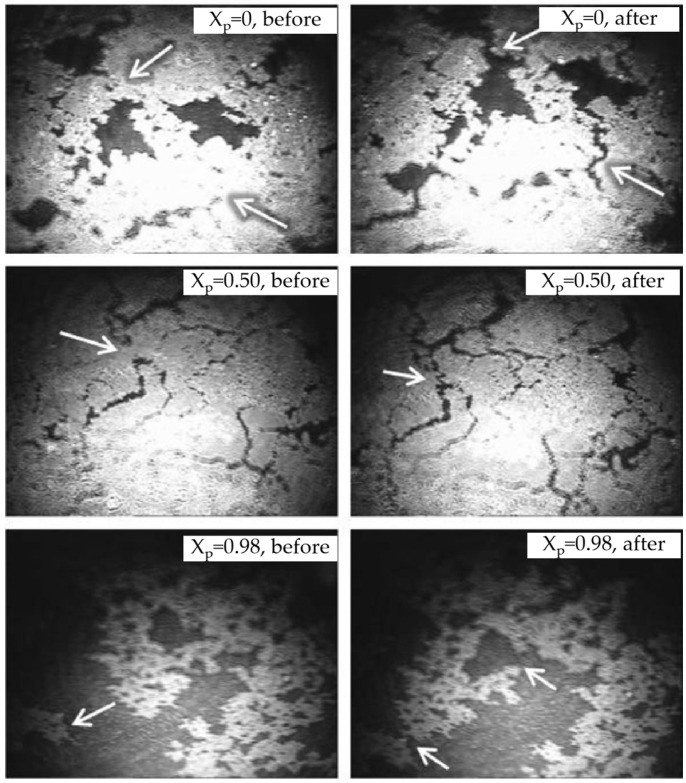
BAM images showing several rafts movements promoted by expansion after monolayer compression for PS-MA-BEE/QDs monolayers of polymer mole fraction. The arrows point to the visible raft movements. Reprinted with permission from Ref. [32]. Copyright 2014, American Chemical Society.

Additionally, Brewster angle microscopy (BAM), a non-contact optical technique for in situ observation and characterization of films at gas–liquid interfaces, including monolayers and nanoparticle layers, is applied to study micro-nanosphere self-assembly. Beatriz Martín-García and colleagues used BAM mainly to observe the macroscopic movement and rearrangement of raft structures in Langmuir monolayers at the air–water interface before and after applying shear stress. As an in situ visualization tool, this technique provides a macroscopic basis for adjusting nanoparticle self-assembly morphologies (Figure 3) [32].

Nevertheless, according to the Abbe equation, optical microscopes are constrained by the optical diffraction limit, precluding the direct resolution of the nanosphere arrangement and individual point defects in sub-100 nm systems [33]. Therefore, optical techniques are generally employed as preliminary screening tools or for mesoscale overview, after which higher-resolution modalities are required for nanoscale structural analysis.

### 3.2. Electron Microscope

Electron microscopy, with spatial resolution generally higher than that of optical microscopy, has become a fundamental tool for characterizing the morphology and structure of nanoscale materials. In the research of nanosphere self-assembly, electron microscopy plays an essential role in assessing assembly quality, identifying lattice defects, and tracing the formation pathways of assembled structures.

#### 3.2.1. Scanning Electron Microscopy (SEM)

The imaging principle of scanning electron microscopy involves using a focused electron beam to scan the sample surface point by point. By detecting signals such as secondary electrons or backscattered electrons emitted from the sample, the signal intensity at each point is converted into brightness, thereby generating a morphological image of the sample surface.

**Figure 4:**
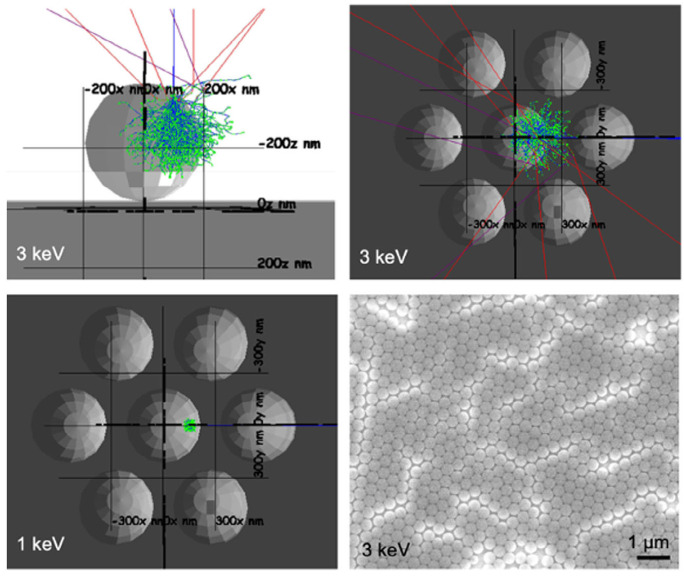
Principle and validation of nanosphere Hexagonal Close-Packing (HCP) defect quantification using SEM edge effects. Reprinted with permission from Ref. [35]. 2020, Wiley-VCH GmbH.

SEM is currently one of the most widely used tools for evaluating the quality of nanosphere self-assembly. Recent reviews indicate that SEM, due to its relatively high spatial resolution, has become an important metrology technique for nanoscale structural characterization and defect inspection [34]. Bekeris et al. employed secondary electron imaging to clearly reveal key features of polystyrene nanosphere monolayers, including the degree of order, lattice defects, missing spheres, and multilayer stacking (Figure 4) [35]. Similarly, Song et al. utilized this technique to characterize self-assembled gold nanosphere structures and calibrated the nanosphere diameter and spacing [36].

These studies highlight the pivotal role of SEM in quantifying defect density and guiding process optimization. Nevertheless, the limitations of scanning electron microscopy include its requirement for a high-vacuum environment, potential sample damage, and relatively low imaging speed. These limitations restrict its use in real-time monitoring of liquid-phase or dynamic processes [37]. In contrast, to elucidate the internal fine structure or dynamic evolution of nanosphere self-assembly at higher resolution, the use of transmission electron microscopy is necessary.

#### 3.2.2. Transmission Electron Microscopy (TEM)

The imaging principle of transmission electron microscopy involves transmitting a high-energy electron beam through an ultrathin specimen. Differences in density, thickness, or crystal structure among various regions of the sample induce changes in the intensity and phase of the transmitted electrons. These are magnified by electromagnetic lenses to generate images of the internal structure.

**Figure 5:**
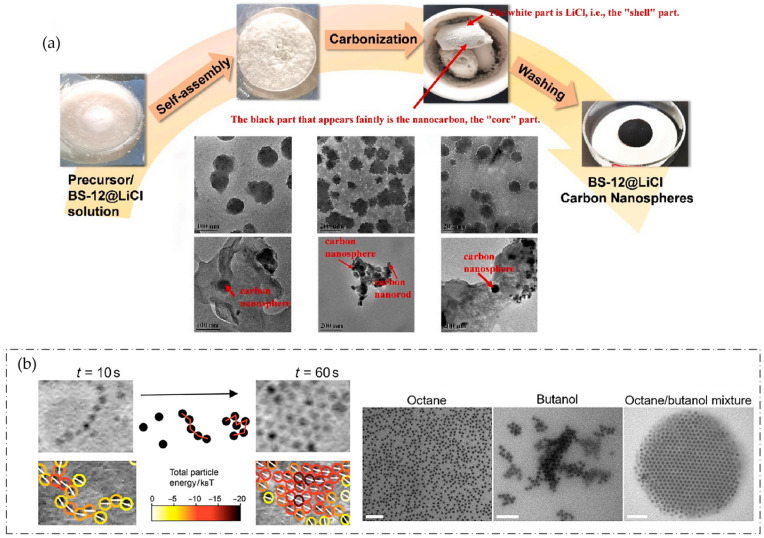
(**a**) Synthesis of carbon nanospheres using surfactant @ salt, including formation mechanism and representative TEM images. Reproduced from Ref. [39]. Copyright 2024, Du, B. et al.; (**b**) in situ TEM dynamic frame images revealing the self-assembly mechanisms and solvent-mediated behaviors of nanoparticles in nonaqueous solutions. Reproduced from Ref. [40]. Copyright 2024, Joodeok Kim et al.

By virtue of its exceptionally high spatial resolution and effective contrast mechanisms, TEM serves as a pivotal technique for elucidating the fine internal architecture, crystal orientation, and atomic-scale defects of self-assembled nanostructures [38]. Du et al. utilized TEM to monitor the entire self-assembly process of core-shell structured carbon nanospheres formed within a surfactant @salt system. From the initial microstructure of precursors to the final morphology of carbonized nanospheres and nanorods, the correspondence between the self-assembly pathway and the resulting structures was distinctly revealed (Figure 5a) [39].

Moreover, liquid-phase transmission electron microscopy (liquid-phase TEM, LPTEM), as an emerging technique, has enabled real-time observation of nanoparticle motion, nucleation, growth, and self-assembly processes within liquid environments (Figure 5b) [40]. This methodology provides an unprecedented perspective for elucidating the dynamic mechanisms underlying defect formation, such as polycrystalline domains and dislocations. Thus, LPTEM serves as a vital bridge between static structural characterization and dynamic processes.

In summary, electron microscopy techniques offer a robust approach for high-resolution characterization of self-assembled nanosphere structures. The significant advantages reside in spatial resolution ranging from sub-nanometer to atomic scale, enabling direct visualization of the assemblies’ morphology, degree of ordering, defect types, and internal fine structures.

Recently, Chen et al. comprehensively reviewed the application of electron microscopy in nanoparticle self-assembly, focusing on advanced characterization techniques like 3D tomography and in situ liquid-cell imaging [41]. In contrast, this review adopts an inspection-oriented perspective specifically for nanosphere assemblies. The defect classification, imaging-system selection, and automated defect detection algorithms, emphasizing the integration of diverse imaging modalities with machine learning for feedback-driven quality control, are systematically bridged.

Despite exceptional spatial resolution, electron microscopy is constrained by the requirement for high-vacuum environments (conventional SEM and TEM) or specialized liquid cells (LP-TEM). The high-energy electron beam may induce irreversible radiation damage in organic or soft-matter nanospheres. Moreover, the comparatively slow imaging speed hampers real-time tracking of rapid dynamic assembly. These limitations have motivated the development of complementary techniques operable under ambient or liquid-phase conditions with minimal sample damage.

### 3.3. Others

Beyond the classical application of optical and electron microscopy, the observation of nanosphere self-assembly structures depends on a range of advanced imaging techniques. These techniques address the limitations of the aforementioned methods by providing chemical specificity, nondestructive three-dimensional detection, or dynamic imaging.

#### 3.3.1. Atomic Force Microscopy (AFM)

AFM is a widely utilized technique based on the principle of scanning a nanoscale tip across the sample surface. By measuring variations in atomic-scale forces, including van der Waals and electrostatic interactions between the tip and the sample, a three-dimensional topographical image of the surface is reconstructed. It can operate under atmospheric, liquid, or specific gaseous atmospheres without requiring conductive treatment of the sample.

Through the tapping mode of AFM, Yilmaz et al. demonstrated that 20–40 nm gold nanoparticles can form continuous, flat, and densely packed monolayers by electric field-assisted self-assembly. Meanwhile, films composed of particles which are smaller than 10 nm tend to display localized vacancies and clustering. Thus, AFM is capable of providing direct morphological evidence of size-dependent assembly behavior [42]. Compared with conventional electron microscopy, the most prominent advantage of AFM is the capacity for in situ and dynamic characterization. High-speed atomic force microscopy (HS-AFM), achieving millisecond temporal resolution, has been successfully employed to capture the real-time growth of supramolecular nanofibers in liquid. It enables direct observation of nanoparticle assembly dynamics and defect evolution at liquid/liquid or solid/liquid interfaces, thereby transforming structural investigations from static end-state analysis to process-visualized studies.

#### 3.3.2. Scanning Near-Field Optical Microscopy (SNOM)

**Figure 6:**
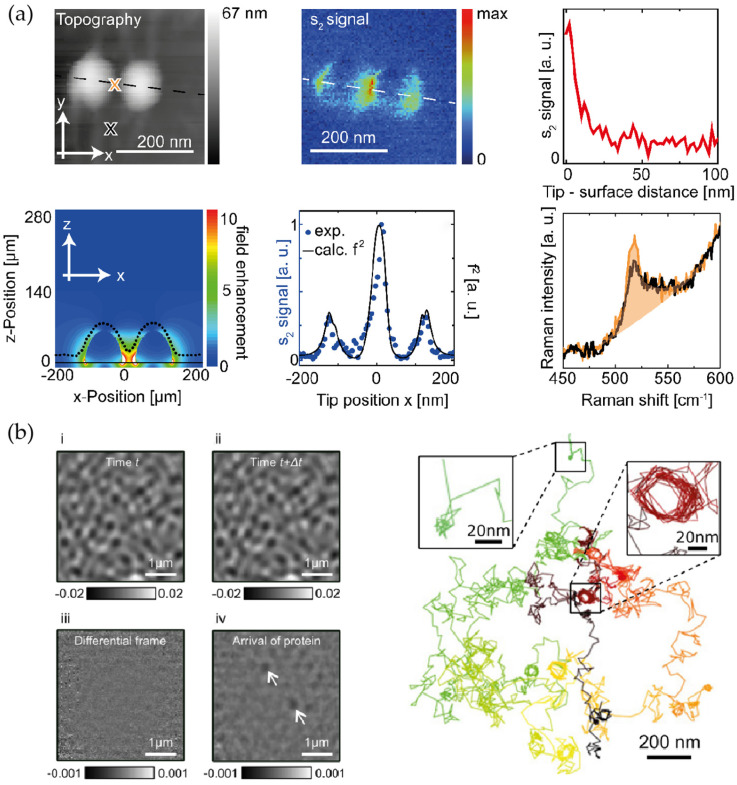
(**a**) Multimodal nanoimaging and analysis of plasmonic hotspots in a 35 nm gap gold nanodimer using sharp silicon tip s-SNOM. Reprinted with permission from Ref. [43]. Copyright 2017 American Chemical Society; (**b**) iSCAT microscopy achieves background suppression via differential imaging, enabling nanoscale dynamic resolution in protein dynamics detection and nanoparticle diffusion tracking on lipid membranes. Reproduced from Ref. [51]. Copyright 2019 American Chemical Society.

SNOM achieves optical resolution of tens to hundreds of nanometers by positioning a tapered optical fiber probe within the near-field region of the sample surface, which is less than 10 nm from the surface. This allows for the collection of evanescent waves or tip-enhanced Raman scattering or fluorescence signals. In the investigation of nanosphere self-assembly, SNOM simultaneously acquires both morphological and near-field optical distributions. It is especially well suited for imaging plasmon modes within assemblies of noble metal nanospheres. Kusch et al. performed correlated nanoimaging of gold nanosphere dimers via scattering-type SNOM. This work directly reveals the spatial distribution of SERS and electromagnetic hotspots. This method still has limitations, which is that the probe is prone to abrasion and has a limited scanning range, and near-field signals are sensitive to surface undulations (Figure 6a) [43].

#### 3.3.3. Complementary Diffraction, Spectroscopic, and Correlative Techniques

Several emerging diffraction, spectroscopic, and correlative imaging modalities can provide additional structural and functional insight into self-assembled nanosphere structures. X-ray-based methods, such as small-angle X-ray scattering (SAXS) and grazing-incidence small-angle X-ray scattering (GISAXS), are highly useful for the nondestructive characterization of lattice symmetry, long-range ordering, interparticle spacing, and domain orientation over statistically representative areas [44,45]. Furthermore, Bragg coherent diffraction imaging (BCDI) utilizes coherent X-ray beams to resolve three-dimensional lattice strain and defect structures within individual super-grains [46]. These methods complement SEM, TEM, and AFM by providing statistically representative or internal structural information that is difficult to obtain from surface morphology alone.

Reflection high-energy electron diffraction (RHEED) is a surface-sensitive technique for characterizing near-surface structures and nanoparticles on substrates [47]. RHEED provides real-time information on surface crystallinity and growth-mode transitions (such as layer-by-layer versus island growth). However, because RHEED strictly requires ultra-high-vacuum conditions and atomically flat substrates, its application is generally limited to the vacuum-based epitaxial growth of inorganic nanocrystals or quantum dot superlattices, rather than wet-chemical colloidal nanosphere assemblies.

Spectroscopic and lifetime-based methods further correlate defects with chemical and optical responses. Micro-Raman spectroscopy can nondestructively probe local strain, crystallinity, phase transformation, stacking disorder, and defect-related vibrational changes, while tip-enhanced Raman spectroscopy (TERS) improves the spatial resolution of such chemical mapping [48]. Fluorescence lifetime imaging microscopy (FLIM) provides spatially resolved fluorescence-decay information, making it useful for evaluating defect states, surface quenching, strain, and energy-transfer effects in luminescent nanospheres, quantum dots, and perovskite nanocrystal supercrystals [49].

To localize and track these structural and functional defects, high-resolution, dynamic, and wide-field optical techniques are further required. Super-resolution optical microscopy techniques (such as STED and DNA-PAINT) utilize fluorescent labeling to extend optical resolution to the nanoscale, enabling the resolution of individual nanospheres and point defects within densely packed arrays under liquid-phase conditions [50]. Interferometric scattering microscopy (iSCAT) operates label-free and can directly detect scattering signals from individual metallic nanospheres at high frame rates, rendering it well suited for real-time tracking of nucleation, migration, and aggregation dynamics of particles at interfaces (Figure 6b) [51]. Surface plasmon resonance imaging employs a wide-field, label-free technique to detect changes in interfacial refractive index, making it suitable for high-throughput monitoring of variations in the surface coverage and adsorption kinetics of nanospheres [52]. Collectively, these techniques address specific requirements such as chemical composition identification, dynamic process visualization, and three-dimensional strain analysis, complementing SEM, TEM, and AFM to establish an integrated multiscale, multi-physical parameter observational framework.

### 3.4. Key Imaging Parameters

The scale and state of the micro-nanosphere self-assembled structures to be observed dictate the choice of imaging system, while the selection and optimization of critical imaging parameters can further satisfy the specific observational requirements.

#### 3.4.1. Resolution and Field of View

Resolution and field of view are often inversely related; achieving high resolution typically necessitates a reduction in the field of view. To mitigate, but not eliminate, this trade-off, scanning imaging methodologies are extensively utilized. Scanning transmission electron microscopy can be implemented either in the TEM equipped with a STEM unit or in the SEM equipped with a STEM detector. In both cases, images are constructed by raster-scanning an electron probe across the sample and collecting transmitted signals point by point, while the accessible field of view, dwell time, signal-to-noise ratio, and effective resolution remain constrained by the underlying instrument and scan strategy. In large-area applications, the observable field of view is generally extended through automated tile acquisition, montage reconstruction, or image stitching, allowing systematic nanoscale observation over broader regions while maintaining a practically controlled, rather than instrumentally maximal, level of resolution [53,54]. Moreover, a multiscale imaging approach, starting with low-magnification global localization followed by high-magnification localized detailed scanning, has demonstrated improved efficiency in the detection of defects over large areas. While these strategies expand the effective imaging area and help reduce sampling bias, they do not fundamentally eliminate the intrinsic trade-off between spatial resolution and field of view.

#### 3.4.2. Temporal Resolution

**Figure 7:**
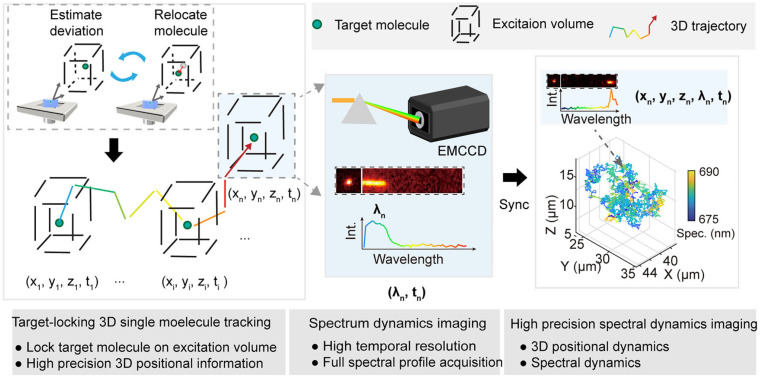
The 3D-SpecDIM system achieves three-dimensional target tracking and synchronization spectral dynamic imaging with high temporal resolution. Reproduced from Ref. [55]. Copyright 2025, Sha, H. et al.

The self-assembly process is intrinsically dynamic. To investigate the mechanisms and progression of defect formation, imaging systems commonly possess high temporal resolution. This is commonly realized through the integration of high-speed cameras and stroboscopic illumination. In the research of rapid self-assembly of colloidal particles at the gas–liquid interface, high-speed imaging at millisecond or even microsecond scales enables the capture of Brownian motion, nucleation, and lattice growth processes. This approach reveals the dynamic factors responsible for dislocation and polycrystalline domain formation. Recent research has increasingly emphasized this field. Recently, the 3D-SpecDIM technique achieved millisecond-scale three-dimensional tracking and synchronous spectral analysis of freely diffusing fluorescent nanospheres in aqueous solutions. Such developments identified instantaneous coupling between rapid nanoparticle movement and local microenvironment spectral variations, advancing the dynamic characterization of self-assembly to a new phase of spatiotemporal chemical multidimensional synchronization (Figure 7) [55].

#### 3.4.3. Contrast Mechanisms in Defect Imaging

Image contrast is the key parameter for distinguishing structural defects from ordered regions. To ensure clarity, contrast mechanisms, which are the physical origins of signal variation, should be distinguished from imaging methodologies, which are the instrumental configurations used to record them. A single contrast mechanism can be shared across multiple imaging modalities, and conversely, one technique may involve several mechanisms. Topographic and height contrast is primarily used to inspect surface packing, vacancies, and grain boundaries. In SEM, this contrast originates from secondary-electron topographic and edge effects, appearing as intensity variations that allow quantification of sphere diameter and separation [56]. AFM complements SEM by providing direct tip–sample height contrast to deliver precise three-dimensional surface profiles [57]. Mass–thickness contrast arises from variations in specimen thickness or density, and is highly effective for identifying particle overlap, voids, and multilayers. This mechanism is shared by various TEM and STEM modalities [41]. For instance, in situ liquid-phase TEM utilizes mass–thickness and projected-density contrast to track vacancy dynamics in liquid environments. By contrast, HAADF-STEM is typically applied to solid or dried assemblies to exploit Z-contrast and thickness-dependent scattering for resolving particle arrangements and interfacial defects. Additionally, diffraction and orientation contrast, arising from differences in crystal orientation or electron channeling, is crucial for revealing grain boundaries and strain in ordered superlattices. Overall, understanding these physical contrast mechanisms provides the essential link between imaging-system selection and defect detection algorithms.

### 3.5. Integration and Intelligence of Imaging Systems

To further enhance observational efficiency and improve the intervenability and manipulability of the self-assembly process, system intelligence and integration are essential.

#### 3.5.1. In Situ Environmental Control Imaging Platform

**Figure 8:**
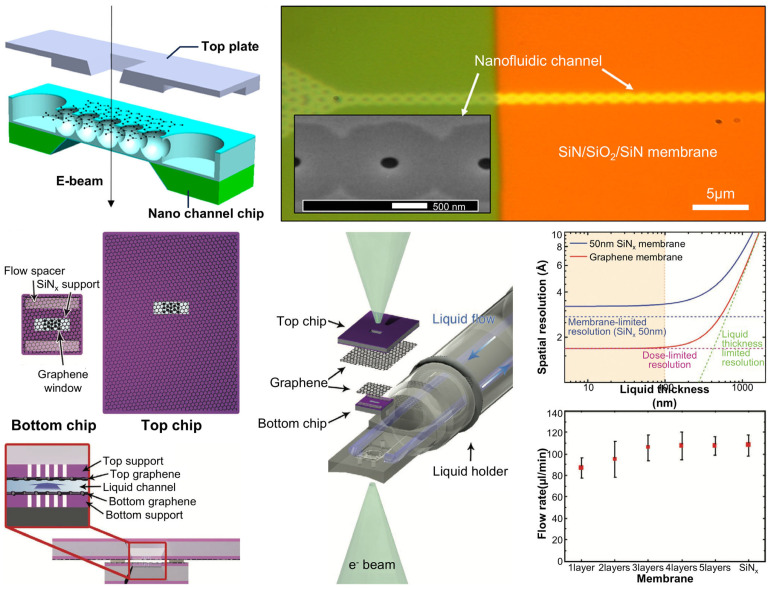
Representative graphene-based and nanofluidic liquid-cell architectures for in situ TEM, including chip configuration, liquid-flow control, membrane design, and spatial-resolution considerations. Reproduced from Ref. [59]. Copyright 2025, Ma, H. et al.

In situ imaging platforms with environmental control refer to imaging systems in which the experimental conditions around the specimen, such as temperature, liquid flow, solvent environment, or reaction medium, are regulated during image acquisition. For nanosphere or nanoparticle self-assembly, these platforms enable direct correlation between externally controlled conditions and structural evolution. Touve et al. employed an integrated temperature-controlled variable-temperature liquid-phase transmission electron microscopy platform. Through applying programmed temperature changes to the solution, they directly observed the complete dynamic process of thermo-responsive block copolymers undergoing polymerization-induced self-assembly into wormlike micelles and vesicles in real time [58]. The graphene liquid cell platform reviewed by Ma et al. integrates flow control capabilities to enable real-time tracking of the Brownian motion and self-assembly aggregation of individual nanoparticles under high-resolution imaging modalities, quantitatively elucidating the joint predominance of monomer attachment and coalescence mechanisms governing nanocrystal growth (Figure 8) [59]. These in situ environmental control platforms directly correlate environmental parameters with structural evolution, transitioning self-assembly observation from static imaging to dynamic analysis, thus providing a technical foundation for the intelligent closed-loop regulation of micro-nanosphere assembly processes.

#### 3.5.2. Machine Vision-Driven Closed-Loop Control System

**Figure 9:**
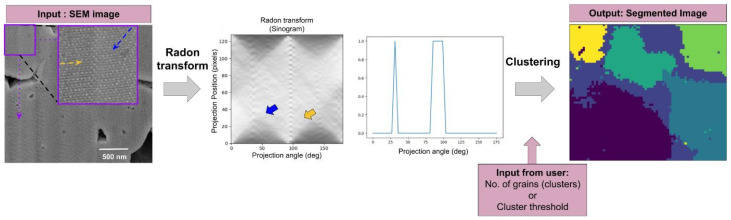
Unsupervised SEM image segmentation workflow for nanoparticle superlattices, where Radon-transform-based orientation features are clustered to identify grains and grain boundaries. Reproduced from Ref. [9]. Copyright 2025, Paruchuri, A. et al.

Image data output by imaging systems serves as inputs for machine learning algorithms and can be utilized to facilitate decision-making processes. As early as 2014, the SelfSys platform realized automated, real-time closed-loop control of nanosphere self-assembly through sophisticated microfluidic chamber design, traceable nanosphere fabrication, high-performance real-time visual tracking, and feedback-based bang-bang control algorithms. In recent years, numerous studies have further advanced the automation and intelligence of nanoscale self-assembly observation by integrating deep learning-driven image analysis with feedback control systems [7]. Ferji et al. developed the Detect-Nano framework grounded in the YOLOv8 deep learning architecture, which automatically identifies and estimates the size of polymer vesicles in TEM images, achieving high-throughput characterization while eliminating bias associated with manual segmentation [10]. Paruchuri et al. utilized the Radon transform in conjunction with unsupervised hierarchical clustering to construct a machine vision workflow that automatically segments SEM images of nanoparticle superlattices, directly producing grain size distribution outputs to facilitate subsequent fabrication processes (Figure 9) [9]. It is evident that the integration of these closed-loop control systems significantly improves the efficiency of recognition and dynamic observation of self-assembled structures.

#### 3.5.3. Multimodal Correlative Microscopy

**Figure 10:**
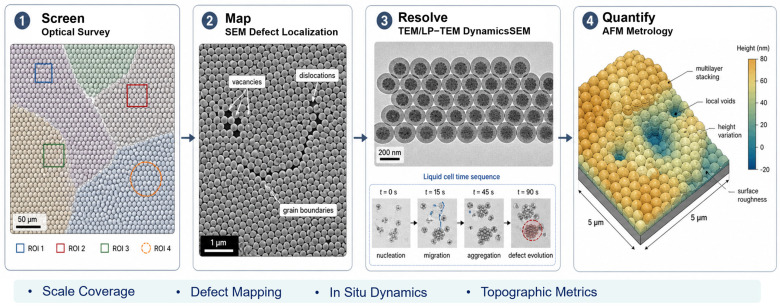
Multimodal correlative microscopy strategy for cross-scale characterization of self-assembled nanosphere structures.

A single imaging modality often proves insufficient to provide comprehensive information; consequently, multimodal correlative microscopy has become a prominent research topic. Dehaerne et al. highlighted in their review the integration of optical microscopy’s rapid screening capabilities with the high-resolution characterization offered by SEM. Initially, optical microscopy is employed for rapid, nondestructive preliminary scanning to localize potential defect regions, which subsequently directs SEM to conduct detailed, high-resolution characterization with a high signal-to-noise ratio in targeted areas [11]. Similarly, integrating the dynamic observational capabilities of liquid-phase TEM with the static high-resolution imaging of SEM facilitates a comprehensive elucidation of defect evolution from inception to final morphology. These correlation strategies balance speed, resolution, and informational dimensions, serving as powerful tools for in-depth analysis of complex defect structures. As summarized in Figure 10, the correlative integration of optical microscopy, SEM, TEM/LP-TEM, and AFM is helpful for constructing a cross-scale imaging workflow for self-assembled nanosphere structures, thereby supporting large-area screening, surface-morphology and defect localization, internal-structure and dynamic assembly analysis, and three-dimensional topographic characterization.

This section systematically presents the imaging technology framework for the self-assembly of nanospheres, covering mainstream methods including optical microscopy, scanning and transmission electron microscopy, atomic force microscopy, and scanning near-field optical microscopy. It analyzes the influence of critical parameters on the visualization of defects within self-assembled structures. Building on this, recent advances in integrated and intelligent imaging are emphasized, including in situ environmental control platforms, machine vision-driven closed-loop control systems, and multimodal correlated microscopy. These strategies collectively enhance the real-time observation capabilities and data dimensionality of dynamic assembly processes. A critical unmet need is the standardization of correlative data pipelines. Currently, image registration between optical, electron, and scanning-probe microscopies is often performed ad hoc, introducing misalignment errors that propagate into subsequent automated defect detection algorithms. Developing open-source, cross-modality alignment tools and associated metadata standards would significantly accelerate the adoption of multimodal workflows.

## 4. Defect Detection Algorithms for Self-Assembled Structures

Defect detection algorithms constitute the pivotal interface between imaging and structural analysis, serving as an essential component for realizing automated quality evaluation and closed-loop feedback control. Their objective is to automatically and precisely identify, localize, and quantify various defects within self-assembled structures from the acquired images. This section systematically delineates defect detection algorithms for self-assembled structures, encompassing image processing, machine learning, and deep learning methodologies, and evaluates the application domains and performance metrics of each approach.

### 4.1. Image Processing Methods

Classical image processing methods, noted for their intuitive principles and independence from large-scale labeled data, are among the first to be applied to defect identification in self-assembled structures. By means of handcrafted feature extraction techniques such as threshold segmentation, morphological operations, edge detection, and frequency-domain filtering, the geometric morphology and spatial distribution of defects can be directly quantified.

#### 4.1.1. Threshold Segmentation

Threshold segmentation, as one of the most fundamental defect detection techniques, relies on grayscale statistics and is suitable for images exhibiting high contrast between background and foreground. This approach is commonly integrated with morphological operations to achieve defect detection. Typically, global or adaptive threshold segmentation is employed initially to separate nanospheres from the background, followed by opening or closing operations to suppress noise and fill voids. Subsequently, connected component analysis is conducted to determine particle count, area, and other metrics, thereby indirectly evaluating defects. Riedl and Lindner applied automated SEM image analysis to nanosphere lithography masks and quantified sphere diameter, sphere–sphere separation, and opening-size distributions. In the workflow, the openings between sphere triples were detected by intensity thresholding [56]. Nevertheless, threshold-based methods exhibit extreme sensitivity to illumination heterogeneity and are limited in effectively addressing low-contrast and complex defect types. For instance, in complex SEM images of nanoparticles with overlapping contours and irregular shapes, conventional threshold-dependent workflows often fail to provide reliable results, whereas deep-learning-based approaches can achieve much more automated, robust, and accurate size and shape analysis [60].

#### 4.1.2. Edge Detection

**Figure 11:**
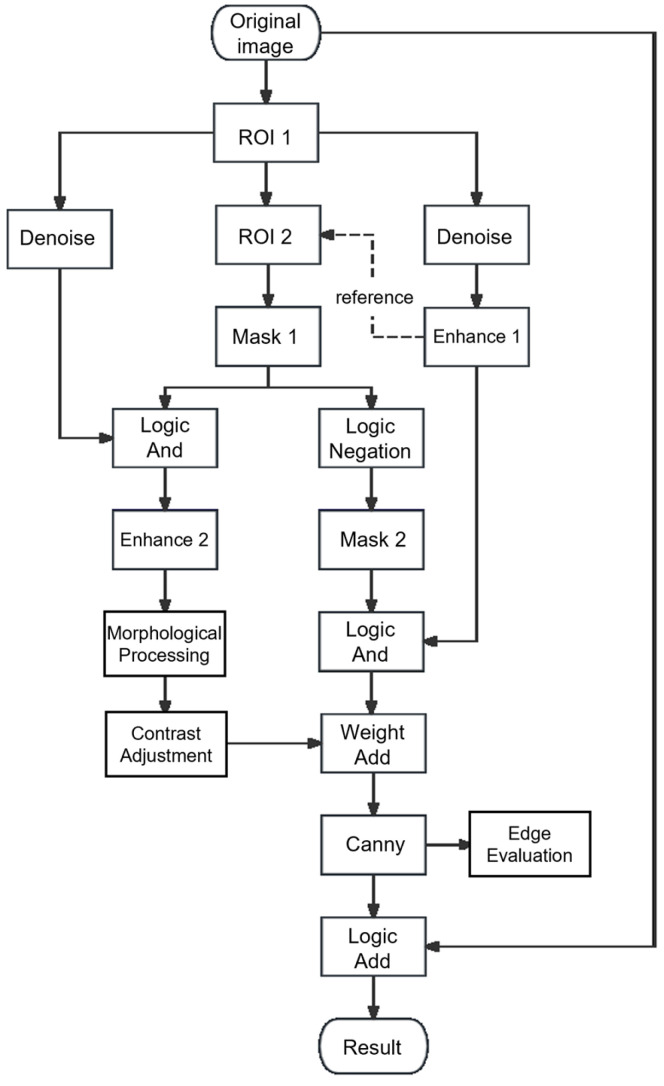
Mask-enhanced SEM edge detection workflow combining denoising, image enhancement, Canny detection, and edge evaluation. Reprinted with permission from Ref. [62]. Copyright 2024, Sun, W. et al.

Edge detection operators are utilized to delineate the contours of individual nanospheres, which are subsequently matched via circular Hough transform or predefined circular templates to precisely determine the center positions of each sphere [61]. In practical applications, Sun et al. address the challenge of blurred multilayer film edges in SEM images by proposing an approach that enhances edge integrity and accuracy through regional segmentation and enhancement algorithms (Figure 11) [62]. In this context, Domonkos et al. developed the HEXI workflow to detect circular nanostructures using Canny edge detection and Hough circle transformation, and to classify them as hexagonally or non-hexagonally ordered based on brightness-variance- or distance-based criteria [18]. Such methods perform effectively under conditions where spherical shapes are regular and images exhibit high clarity, analogous to thresholding techniques. However, they involve high computational complexity and degrade significantly for overlapping, deformed, or blurred spheres.

#### 4.1.3. Frequency-Domain Analysis

**Figure 12:**
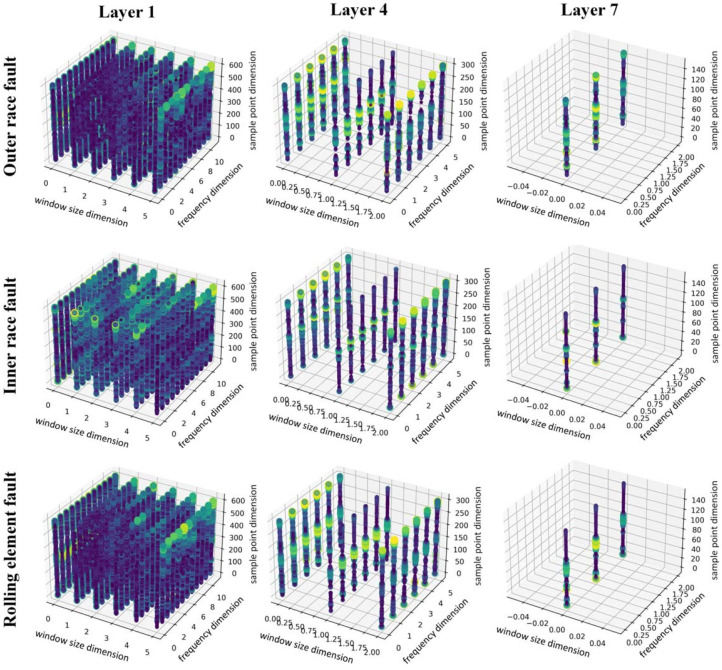
Multi-resolution short-time Fourier transform (STFT) feature maps extracted by a 3D CNN, illustrating layer-dependent representations for outer-race, inner-race, and rolling-element fault classification. Reprinted with permission from Ref. [65]. Copyright 2024, Zhang, M. et al.

In contrast to threshold segmentation and edge detection, frequency-domain methods leverage the long-range periodicity inherent in self-assembled nanosphere arrays to extract defect-related information within the transform domain. A well-ordered lattice produces distinct Bragg peaks in its Fourier transform (FFT), whose sharpness, intensity distribution, and higher-order diffraction characteristics provide a global metric of ordering and an indirect estimate of defect density [63]. Beyond long-range periodic lattices, Cesaria and co-workers used autocorrelation-assisted FFT analysis to quantify short-range ordering in nanohole arrays and emphasized that the nearest-neighbor distance should not be directly treated as the lattice constant of a periodic array [20,21]. Krämer et al. (2023) apply coherent Fourier scattering measurement techniques to detect arrangements of gold nanospheres, discriminating different configurations and orientations below the diffraction limit by analyzing Fourier-plane intensity distributions, which facilitated rapid identification of particle patterns [64]. For example, Zhang converted one-dimensional rolling-bearing vibration signals into multi-resolution short-time Fourier transform (STFT) representations and used a 3D CNN to classify different fault types (Figure 12) [65]. Although this work does not directly address microscopy images or nanosphere self-assembly defects, it illustrates a transferable strategy: multi-resolution frequency-domain features can help neural networks capture scale-dependent periodic disturbances. Therefore, frequency-domain filtering is frequently utilized as a preliminary preprocessing step to eliminate periodic backgrounds or suppress noise, providing cleaner images for subsequent edge detection or threshold-based segmentation.

These approaches constitute classical image processing methods, addressing problems from different perspectives, ranging from spatial domain to transform domain, and from regional grayscale analysis to global frequency analysis, thereby complementing each other. Nevertheless, these methods share inherent limitations in that their performance degrades precipitously under uneven illumination, particle agglomeration, or complex defect topologies, necessitating more adaptive, data-driven strategies.

### 4.2. Machine Learning Methods

To overcome the limitations of image processing methods regarding generalization capability and automation level, researchers have introduced machine learning approaches. These methods extract multidimensional handcrafted features encompassing geometric, texture, and frequency-domain attributes from images, which are then used as input for classifier training to enable automatic defect identification and classification. This section systematically reviews the application and performance of representative machine learning algorithms, including support vector machines, Random Forests, and unsupervised techniques, in the detection of defects within self-assembled structures.

#### 4.2.1. Support Vector Machine (SVM)-Based Methods

Support vector machines construct a mapping from a feature space to defect categories by extracting multidimensional handcrafted features from images, exhibiting robust generalization capability in small-sample classification tasks. Kurbakov et al. extracted seven interpretable features of nanoparticles from SEM images and utilized a linear SVM to achieve classification performance comparable to that of deep neural networks [66]. The aforementioned research demonstrates that support vector machines combined with interpretable feature engineering can effectively support the analysis of structural order in self-assembled particle distribution and particle identification under noisy conditions. Despite this, its capacity to model nonlinear feature relationships remains limited. Random Forests and ensemble learning methods could provide a robust solution to this limitation.

#### 4.2.2. Random Forest and Ensemble Methods

**Figure 13:**
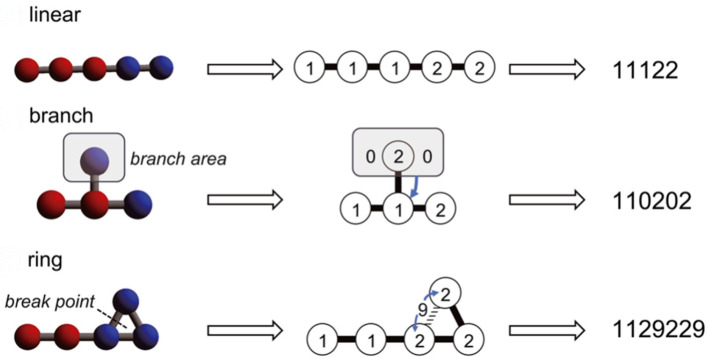
Modified-SMILES encoding of coarse-grained amphiphilic molecular models, showing the conversion of linear, branched, and cyclic structures into numerical descriptors for machine learning prediction of self-assembly behavior. Reprinted with permission from Ref. [67]. Copyright 2024, Ishiwatari, Y. et al.

Random Forest (RF), as a prototypical ensemble learning algorithm, constructs multiple decision trees and aggregates their predictions through voting or averaging. This approach effectively manages small sample sizes, high-dimensional feature spaces, and complex nonlinear relationships, exhibiting strong performance in predicting defect growth and structural characteristics of nanosphere self-assembly. Ishiwatari et al. employed Random Forest to predict critical packing parameters from the SMILES representations of 305 amphiphilic molecules. The model’s predictive performance (R^2^ > 0.9) significantly surpassed that of linear regression. Feature importance analysis revealed that the ratio of hydrophilic to hydrophobic moieties constitutes a key factor determining self-assembled structures such as micelles and vesicles (Figure 13) [67]. For in situ tracking of self-assembly growth, researchers generated morphological images of carbon nanotube forests at various growth stages using physical numerical simulations. After extracting histogram texture features, a Random Forest classifier was constructed, effectively predicting the growth parameters [68]. RF can effectively integrate multi-source features, such as molecular structure encoding and image texture, demonstrating high accuracy and interpretability in both the prediction of self-assembled morphologies and the in situ analysis of growth processes. This provides robust support for the controllable design of nanoscale self-assembled structures.

#### 4.2.3. Unsupervised Techniques and Complementary Methods

**Figure 14:**
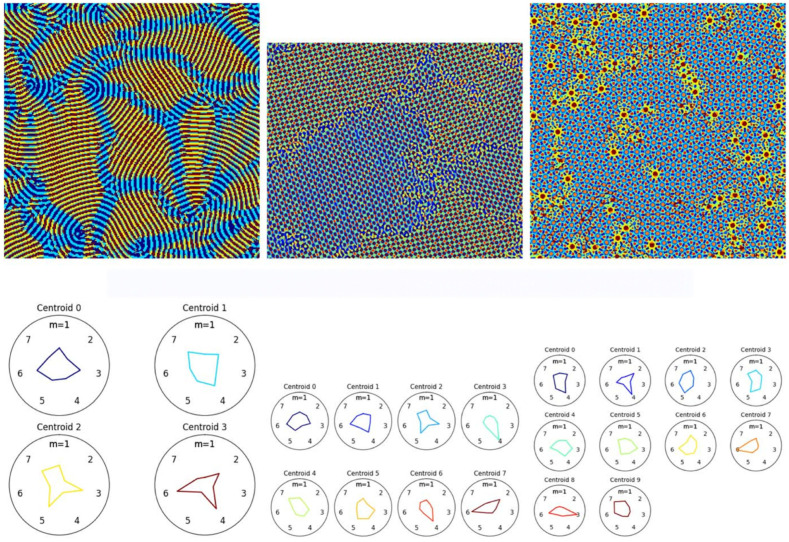
Unsupervised k-means clustering for decomposing shapelet response vectors into k centroid vectors. Reprinted with permission from Ref. [69]. Copyright 2024, Tino, M. et al.

Beyond the previously mentioned supervised learning methods exemplified by SVM and Random Forests, unsupervised clustering and dimensionality reduction techniques offer complementary avenues for exploring defects in self-assembled structures. The unsupervised methods in this context depend on manually engineered or physically extracted features as inputs, rather than learning feature representations end-to-end directly from raw images. Tino et al. proposed an image analysis approach based on Shapelet functions, employing these basis functions for dimensionality reduction applied directly to SEM, AFM, and TEM images of self-assembled surfaces. This method enables pixel-level local orientation vectorization and the identification of topological defects without requiring prior knowledge of ordered symmetries. It effectively handles orientation defects and domain boundaries across various self-assembled textures, including stripe and hexagonal patterns (Figure 14) [69]. In scenarios characterized by scarce annotated data or unknown defect types, unsupervised methods can reveal anomalous patterns within self-assembled structures through feature space projection or basis function decomposition, offering a technical approach to defect detection distinct from supervised learning.

Aside from the aforementioned supervised and unsupervised strategies, a range of less commonly employed yet complementary approaches have further expanded the utility of machine learning for defect detection in self-assembled nanostructures. Spectral clustering can be used as a graph-based segmentation strategy to partition pixel-similarity graphs, which is helpful for detecting spatially continuous defects such as grain boundaries [70]. Gaussian mixture models can classify local particle environments by modeling distributions of structural descriptors, such as bond-orientational order parameters [71]. Active learning can iteratively select uncertain or underrepresented samples for annotation, thereby reducing labeling costs while improving the coverage of rare defect categories [72]. The three methods address the limitations of the previously mentioned approaches in region segmentation, statistical outlier detection, and optimization of annotation efficiency. In spite of this, all remain constrained by the representational capabilities of manual features when characterizing complex defect morphologies. Consequently, this necessitates the progression from manual feature engineering to end-to-end feature learning via deep learning.

### 4.3. Deep Learning Methods

In contrast to traditional machine learning approaches that depend on manual feature engineering, deep learning employs deep multilayer neural networks to automatically learn hierarchical features directly from raw images, thereby exhibiting superior representational capacity and generalization performance in defect detection and recognition tasks conducted under complex backgrounds. With respect to the observation of nanosphere self-assembly structures, deep learning methods have primarily evolved along two technical approaches: the first involves pixel-level defect detection based on semantic segmentation, which aims to accurately delineate the boundaries and morphology of defects; the second focuses on defect recognition and localization utilizing object detection techniques, emphasizing rapid identification of defect regions and the output of their spatial coordinates. This section reviews the advancements of these two methodological categories in characterizing self-assembled structures.

#### 4.3.1. Pixel-Level Semantic Segmentation

Semantic segmentation assigns categorical labels to each pixel, enabling precise pixel-level localization of defect regions. This approach is particularly effective in complex scenarios within nanosphere self-assembly structures, where defect boundaries are blurred, shapes are irregular, and contrast with surrounding normal regions is low. In semiconductor defect detection, instance segmentation networks such as Mask R-CNN have been successfully applied to SEM images. That can generate accurate pixel-level masks for various defect types, including bridging, breakage, and linewidth collapse, and facilitate quantification of their area and frequency [73]. Roberts et al. developed DefectNet, a deep convolutional neural network for semantic segmentation of line dislocations, precipitates, and voids in diffraction-contrast STEM images of structural alloys. Although the investigated system was not a self-assembled nanosphere structure, this work demonstrated the potential of pixel-level deep-learning segmentation for quantitative defect analysis in advanced microscopy images [74]. Semantic segmentation techniques enable precise identification and quantitative analysis of defects within micro-nanosphere self-assembly structures at the pixel or voxel scale, thereby providing essential technical support for the in situ characterization of the self-assembly process.

#### 4.3.2. Object Detection for Defect Recognition and Localization

Unlike semantic segmentation, which aims for pixel-level fine classification, object detection methods employ bounding boxes to annotate the positions and categories of defect regions, offering higher computational efficiency while maintaining detection accuracy. This approach is particularly suitable for the rapid identification and localization of defects in nanosphere self-assembled structures. Typical deep learning object detection frameworks include the two-stage Faster R-CNN, the single-stage YOLO series, and the Transformer-based DETR. Recent studies have demonstrated that adopting the Faster R-CNN framework, utilizing its region proposal network (RPN) for shared convolutional features, enables pattern-independent defect detection on SEM images. This achieves a mean average precision (mAP) of 83% at an intersection over union (IoU) threshold of 0.5, significantly reducing the time required for manual inspection [75]. In the analysis of TEM images of self-assembled nanostructures, Ferji et al. proposed the Detect-Nano detection framework based on YOLOv8, incorporating a weighted bounding box fusion strategy to enable automatic detection and size estimation of polymer vesicles, demonstrating strong generalization performance on unseen TEM images [10].

It is important to note that the aforementioned methods all depend on large-scale pixel-level or bounding box-level annotated datasets, and the high cost of obtaining such annotations remains a significant bottleneck for the application of deep learning in defect detection. Self-supervised learning, by designing pretext tasks, acquires general feature representations from unlabeled images, thereby substantially reducing the reliance on annotated samples for downstream tasks. SimCLR, for instance, demonstrates that contrastive representation learning can substantially improve label efficiency in general image-recognition tasks; when fine-tuned with only 1% of ImageNet labels, it achieves 85.8% Top-5 accuracy [76]. Weakly supervised learning employs image-level labels to train models and utilizes class activation mapping methods such as Grad-CAM to localize defect regions, thus avoiding the laborious process of pixel-level annotation. Recent research has successfully applied these strategies to defect detection in semiconductor wafers and SEM images [11,77]. For the observation of nanosphere self-assembled structures, these methods can greatly reduce the amount of manual annotation, particularly suited to complex scenarios characterized by diverse defect morphologies and blurred boundaries. They constitute a highly promising cutting-edge direction in this field.

This section provides a sequential review of three categories of defect detection methods for self-assembled nanosphere structures from the perspective of methodological development. Traditional image processing methods depend on manually designed rules, such as edge detection and threshold segmentation, and are most suitable for simple scenarios with high contrast. Traditional machine learning methods achieve good interpretability under limited sample conditions by combining manually extracted features with classifiers. Deep learning methods automatically learn hierarchical features in an end-to-end fashion and demonstrate excellent performance in pixel-level segmentation and rapid localization of complex defects. Each category has its specific application scenarios, and collectively, they form the fundamental methodological framework for defect detection in self-assembled structures. Despite impressive progress, deep learning models for colloidal defect detection remain material-specific and modality-specific. A model trained on SEM images of silica nanospheres rarely generalizes to TEM images or polystyrene assemblies without extensive retraining. The field critically lacks public benchmark datasets spanning multiple particle compositions, sizes, and imaging conditions. Future work should prioritize physics-informed neural networks that encode crystal symmetry as inductive biases, domain-adversarial training for cross-modality transfer, and synthetic-to-real transfer learning using molecular dynamics or Monte Carlo simulations to generate labeled training data with realistic noise models.

## 5. Conclusions

This review summarized imaging systems and defect detection algorithms for self-assembled nanosphere structures. It discussed representative self-assembled architectures and common defect types, including vacancies, interstitial particles, dislocations, grain boundaries, multilayer stacking, voids, and cracks. These defects can disrupt structural order and compromise the reliability of optical, mechanical, and functional performance. The review then elucidated major imaging modalities used for structural characterization and defect inspection. Optical microscopy is well suited for rapid, nondestructive, and large-area screening, while SEM and TEM provide higher spatial resolution for observing surface morphology, lattice ordering, internal structures, and local defects. AFM, SNOM, and other complementary techniques can further provide three-dimensional topographic information and localized optical responses. In addition, in situ environmental control, machine-vision-based feedback, and multimodal correlative microscopy are advancing imaging systems beyond static post-characterization toward dynamic and intelligent analysis. In terms of algorithmic analysis, classical image processing methods, such as threshold segmentation, edge detection, and frequency-domain analysis, remain useful for simple and high-contrast images. Machine learning methods improve feature-based classification and pattern recognition, while deep-learning-based segmentation and object detection methods show strong potential for automated, high-throughput, and reproducible defect localization. Overall, reliable defect inspection of self-assembled nanosphere structures requires the coordinated development of imaging hardware, image quality control, algorithmic robustness, and quantitative evaluation strategies.

Future research should focus on standardized, adaptive, and feedback-driven inspection workflows. First, open benchmark datasets are needed. Such datasets should cover different materials, particle sizes, assembly morphologies, imaging modalities, magnifications, and defect types, while also containing reliable annotations and clear metadata. They would support fair comparison among algorithms and improve model transferability. Second, multimodal correlative microscopy requires more robust image registration and cross-modality alignment tools. Optical, electron, and scanning-probe images should be linked quantitatively across different length scales, which also requires shared metadata standards and reproducible data-processing pipelines. Third, data-efficient learning methods should be further developed. Transfer learning, semi-supervised learning, self-supervised learning, synthetic data generation, and domain adaptation may reduce the need for extensive manual annotation [10,11]. These methods may also improve robustness across different imaging conditions and defect morphologies. In addition, physics-informed models could be introduced to incorporate lattice symmetry, particle-packing rules, assembly kinetics, and defect-evolution mechanisms. This strategy may improve both interpretability and reliability. Finally, real-time imaging, automated defect detection, and process feedback should be more closely integrated. Such integration may enable closed-loop optimization of nanosphere self-assembly. In this way, defect information could be used not only for post-characterization, but also for guiding assembly conditions and improving structural quality. Ultimately, the integration of advanced imaging systems, intelligent defect detection algorithms, and closed-loop process control will be essential for advancing nanosphere self-assembly toward more reliable, scalable, and application-oriented nanomanufacturing.

## Data Availability

No new data were created or analyzed in this study. Data sharing is not applicable to this article.
